# Dissecting the details: A case-based anatomical walkthrough of the antegrade posterior interosseous artery flap for elbow reconstruction

**DOI:** 10.1016/j.jpra.2025.11.008

**Published:** 2025-11-14

**Authors:** Andrea Sól Kristjánsdóttir, Anna Duncan, Eleonora O.F. Dimovska

**Affiliations:** a Department of plastic and maxillofacial surgery, Uppsala University Hospital, Sjukhusvägen 85, 751 85 Uppsala, Sweden; b Department of Surgical Sciences Uppsala University Hospital, Sjukhusvägen 70, 751 85, Uppsala, Sweden; c Division of Plastic Surgery, Dalhouise University, Saint John, New Brunswick, E2L4L5, Canada

**Keywords:** Elbow reconstruction, Upper extremity trauma, Antegrade posterior interosseous artery flap, Posterior interosseous nerve, Pedicled flap

## Abstract

The pedicled posterior interosseous artery flap is a reliable and effective option for reconstructing soft tissue defects in the upper extremity. Commonly described in a retrograde fashion for distal defects, its antegrade counterpart is less frequently reported, particularly in relation to elbow reconstructions. Additionally, there are few and less detailed descriptions on how to safely and correctly identify the posterior interosseous nerve (PIN) in the harvest. The current case describes the use of the pedicled antegrade posterior interosseous artery flap to resurface a complex elbow wound with exposed triceps tendon, exposed ulnar nerve and an open elbow joint. We present a detailed step-by-step harvest with the additional anatomical description of the topography of the PIN branches relative to the PIA pedicle.

## Introduction

The elbow presents a challenging reconstructive area as the demands of its hinge-joint range of movement requires pliable, but robust soft tissue coverage.^1^ The pedicled posterior interosseous artery (PIA) flap has been commonly described in a retrograde fashion for reconstructing distal defects on the hand and wrist, but is equally an excellent option for proximal defects in the forearm or elbow.^2^ This short communication describes the use of the antegrade PIA flap to reconstruct a traumatic soft-tissue elbow wound, in a detailed step-by-step approach.

## Case

A 56-year-old male presented with a 10 × 7 cm traumatic elbow wound with an open joint and exposed triceps tendon and ulnar nerve, following a motorcycle accident. Radiographs of the upper extremity had excluded any fractures. Due to other concomitant injuries, reconstruction of the wound was delayed, allowing for partial healing by secondary intention. The wound was debrided in theatre, underwent serial bedside debridements and was temporized with a vacuum assisted closure (VAC). The bone was infection free, and the ulnar nerve was intact and not exposed at the time of reconstruction.

## Surgical technique

### Pre-operative markings

**Figure 1:**
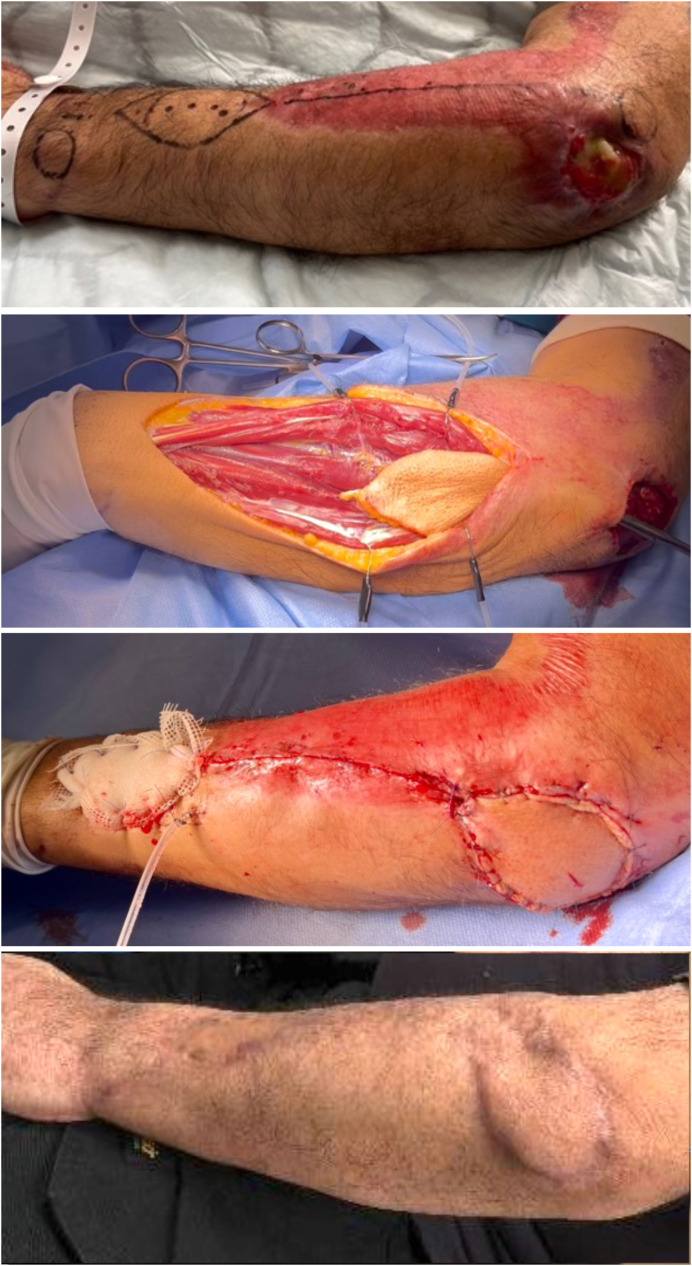
Demonstrating preoperative markings of the axis between the lateral epicondyle (proximal) and the distal radioulnar joint with the designed skin paddle and doppler identified perforators, tunneling of the flap into the defect, flap inset after division of the subcutaneous tunnel and follow-up of the flap and donor site at 12 months post-operatively.

Markings are completed as per Zaidenberg et al.^2^ The elbow is flexed to 90 degrees with the wrist fully pronated. The axis between the lateral epicondyle and the distal radioulnar joint (DRUJ) is drawn. The skin paddle is designed as an ellipse, distally along this axis, overlying doppler-identified perforators (Figure 1).

### Harvesting technique

**Figure 2:**
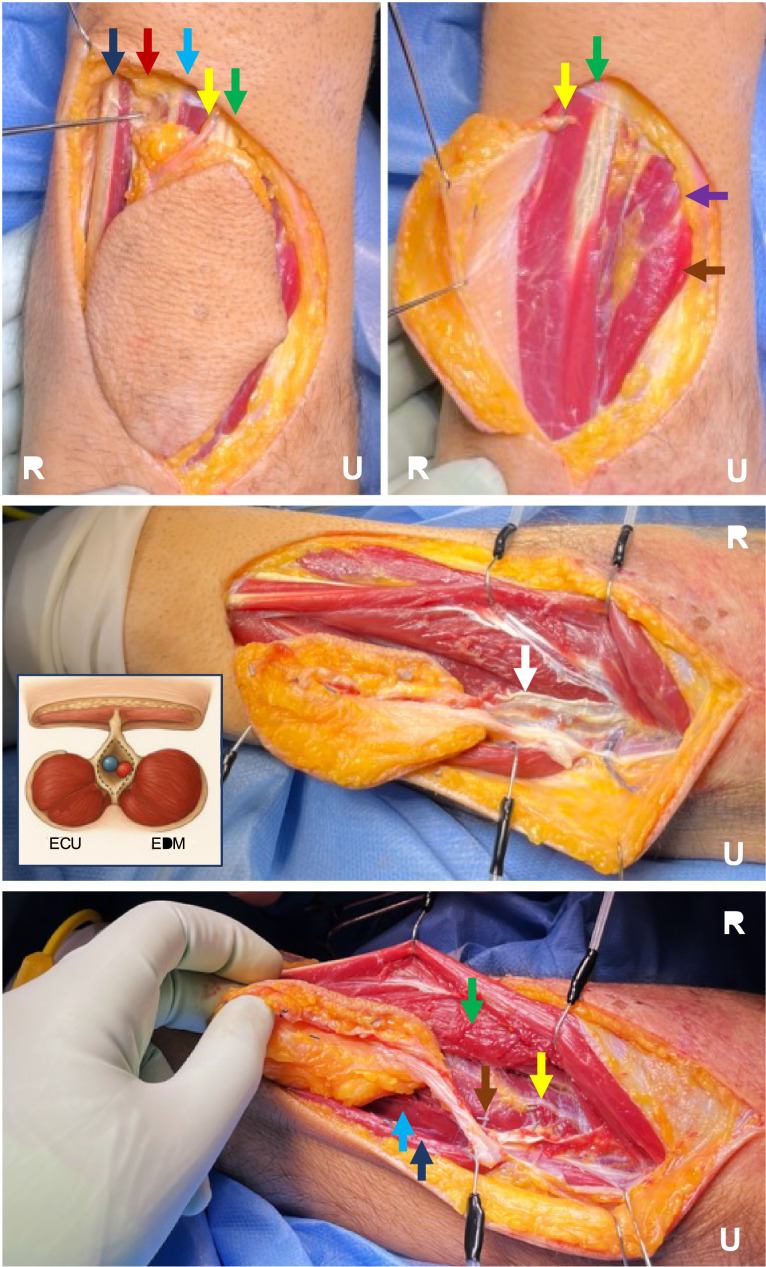
Showing a circumferential subfascial skin incision, elevation of the flap, harvest of pedicle with inclusion of the septal and muscular fascias (with an additional AI generated image showing inclusion of the septum and muscular fascias with the pedicle) and exposure of the posterior interosseous nerve after retraction of the extensor digitorum. R: Radial, U: Ulnar, ECU: Extensor carpi ulnaris, EDM: Extensor digiti minimi. Arrows –Dark blue: Extensor carpi ulnaris, Red: Posterior interosseous vascular pedicle, Blue: Extensor digiti minimi, Yellow: Posterior cutaneous antebrachial nerve of the forearm, Green: Extensor digitorum, Purple: Extensor pollicis brevis, Brown: Abductor pollicis longus, White: Muscular fascia.

Under tourniquet control, flap harvest is performed subfascially around the skin paddle and is elevated from radial to the ulnar. Perforators from the PIA pedicle are identified emerging through the septum between the extensor carpi radialis (ECU) and extensor digiti minimi (EDM) (Figure 2). The posterior antebrachial cutaneous nerve (PACN) of the forearm is encountered subcutaneously distal to the skin paddle and can be used for sensate reconstructions. Elevation of the skin paddle reveals the underlying muscles of abductor pollicis longus (APL), extensor pollicis brevis (EPB), extensor digitorum (ED), extensor digiti minimi(EDM) and extensor carpi ulnaris (ECU). The EDM is retracted radially to visualize the septum between ECU and EDM, and the ECU is retracted ulnarly to isolate the skin paddle over the septum. The distal continuation of the PIA is ligated, allowing the septum and skin paddle to be dissected free from distal to proximal. As the dissection proceeds proximally, small muscle branches are ligated and care is taken to preserve the fascial septum and fascias from adjacent muscles, so as not skeletonize the pedicle (Figure 2). Distal branches of the posterior interosseous nerve (PIN) are visualized on the surface of abductor pollicis longus (upon retraction of extensor digitorum) and protected. The dissection proceeds proximally to the point of rotation at the take-off of the PIA from the interosseous trunk, typically 6 cm from the lateral epicondyle. The supinator can be split to gain additional pedicle length. Following tourniquet let-down and hemostasis, the flap is delivered to the defect through a subcutaneous tunnel (preferably split to prevent pedicle compression) (Figure 1). The donor site is covered with a full thickness skin graft and the flap is sutured loosely to eliminate tension.

### Post-operative management

Postoperatively, a cast was used to immobilize the elbow at 90° of flexion to prevent kinking or tension of the pedicle. Daily flap monitoring was conducted through a window in the dressing. On postoperative day 5, the cast was removed, gradual movement of the elbow was initiated and the patient was discharged home. During the 1-month follow-up visit the patient had obtained full ROM of the elbow. No complications or adverse outcomes were noted at a final 12 months follow up.

## Discussion

The choice of reconstructive techniques of the elbow aim to retain its wide hinge-joint range of movement and provide robust, yet thin and pliable coverage to facilitate early mobilization and rehabilitation to prevent stiffness and functional degeneration.^4^ The PIA flap has been reported a good option in this regard, along with the benefit of avoiding sacrificing a major artery to the hand and having the ability to provide additional fascia for lining of the elbow joint when necessary.^1^^,^^2^^,^^5^ Ciftci et al. compared the use of lateral arm flap and PIA flap for elbow and reported significantly better functional outcomes along with shorter operating time for patients receiving a PIA flap.^1^ However, potential drawbacks include the need to skin graft the donor site, potential injury to PIN nerve branches and limited coverage of larger defects.^1^^,^^5^ Furthermore, performing sensory nerve coaptation of the PACN of the forearm is recommended to improve sensory restoration and limit functional trauma at the elbow.^3^

## Conclusion

The antegrade PIA flap is a good option for elbow reconstruction, especially where the repair of the joint capsule cannot be achieved. The PIA flap provides durable wound coverage with the potential for sensory reinnervation and enables early rehabilitation, thereby decreasing morbidity and improving functional outcomes.

## Consent

Written informed consent was obtained from the patient for publication of this case report and accompanying images. A copy of the written consent is available for review by the Editor-in-Chief of this journal on request.

## Funding

None.

## Ethical approval

Not required.

## Declaration of competing interest

The authors report no conflicts of interest regarding this case report.

## References

[1] Çiftci S. (2023). Comparison of lateral arm flap and posterior interosseous artery flap for soft tissue reconstruction of the elbow. Ulus Travma Ve Acil Cerrahi Derg.

[2] Zaidenberg E.E., Zancolli P., Farias Cisneros E., Miller A.G., Moreno R. (2018). Antegrade posterior interosseous flap for nonhealing wounds of the elbow: anatomical and clinical study. Plast Reconstr Surg Glob Open.

[3] Kamei W., Murakami M., Nakamoto K. (2021). Spare parts surgery with a free posterior interosseous artery perforator flap for thumb tip. Plast Reconstr Surg Glob Open.

[4] Nakao J., Umezawa H., Ogawa R., Mateev M.A. (2018). Reconstruction of elbow skin and soft tissue defects using perforator-pedicled propeller flaps. Microsurgery.

[5] Robinson L.P., Usmani R.H., Hazel A., Gupta A. (2021). Use of the antegrade posterior interosseous artery flap for coverage of complex elbow wounds. Plast Reconstr Surg.

